# Development of a quantitative preclinical screening model for implant osseointegration in rat tail vertebra

**DOI:** 10.1007/s00784-018-2661-1

**Published:** 2018-10-29

**Authors:** Sándor Farkasdi, Dávid Pammer, Róbert Rácz, Gergely Hriczó-Koperdák, Bence Tamás Szabó, Csaba Dobó-Nagy, Beáta Kerémi, József Blazsek, Frederic Cuisinier, Gang Wu, Gábor Varga

**Affiliations:** 1 Department of Oral Biology, Semmelweis University, Nagyvárad tér 4., Budapest, 1089 Hungary; 2 Department of Materials Science and Engineering, Budapest University of Technology and Economics, Budapest, Hungary; 3 Department of Oral Diagnostics, Semmelweis University, Budapest, Hungary; 4 Laboratoire Bioingénierie et Nanosciences EA4203, Université Montpellier, Montpellier, France; 5 Oral Implantology and Prosthetic Dentistry, Academic Centre for Dentistry Amsterdam (ACTA), Vrije University Amsterdam and University of Amsterdam, Amsterdam, The Netherlands

**Keywords:** Osseointegration, Implant, Micro-CT, Pull-out test, Resonance frequency analysis, Histomorphometry

## Abstract

**Objectives:**

Functional tooth replacement and bone regeneration are parts of the daily practice in modern dentistry, but well-reproducible and relatively inexpensive experimental models are still missing. We aimed to develop a new small animal model to monitor osseointegration utilizing the combination of multiple evaluation protocols.

**Material and methods:**

After cutting the tail between the C4 and C5 vertebrae in Wistar rats, costume made, parallel walled, non-threaded implants were placed into the center of the tail parallel with its longitudinal axis using a surgical guide. Osseointegration of the titanium implants was followed between 4 and 16 weeks after surgery applying axial extraction force, and resonance frequency analysis as functional tests, and histomorphometry and micro-CT as structural evaluations.

**Results:**

In functional tests, we observed that both methods are suitable for the detection of the time-dependent increase in osseointegration, but the sensitivity of the pull-out technique (an approximately five times increase with rather low standard error) was much higher than that of the resonance frequency analysis. In structural evaluations, changes in the detected bone implant contact values measured by histomorphometry (yielding 1.5 times increase, with low variations of data) were more reliable than micro-CT based evaluations to screen the developments of contact between bone and implant.

**Conclusion:**

Our results provide evidence that the caudal vertebrae osseointegration model is useful for the preclinical evaluation of implant integration into the bone.

**Clinical relevance:**

The combination of the biomechanical and structural tests offers a well-reproducible small animal system that can be suitable for studying the integration of various implant materials and surface treatments.

## Introduction

Since osseointegration was introduced by Branemark as a rigid fixation of an implant within bone tissue more than half a century ago [1], numerous in vitro, preclinical and clinical studies have been carried out to investigate this process. Osseointegration is defined as the direct structural and functional contact between the artificial implant surface and the living bone tissue [2]. Furthermore, the term also refers to the process of forming this direct fixation which has high dependency on the preceding surgical procedure and preoperative circumstances [3]. The process starts when the primary stability of the implant is achieved by mechanical fixation [4]. Then, bone regeneration and remodeling proceed continuously, which finally leads to a rigid and stable fixation of the implant into the surrounding bone tissue. After the initial bone healing around titanium implants, bone remodeling is practically lifelong [5]. In spite of continuous efforts, the course of osseointegration, bone remodeling, and regeneration around the implants has not yet been fully understood [6, 7]. To more extensively investigate this complex process, there is a need to develop reliable and reproducible preclinical and clinical methods.

Implant stability, an important indicator for the level of osseointegration can be assessed in both invasive and non-invasive ways. Non-invasive methods include radiological analysis/diagnostics [8], resonance frequency analysis (RFA) [9], “damping characteristics” [10], and also the perception of surgeons [11]. Invasive techniques include pull-out/push-in test [11–13], reverse/removal torque measurement [14], cutting torque resistance [15], seating torque [16], tensional force [17], micromotion testing [18], and histology/histomorphometry [19–21]. Invasive methods are not applicable for clinical monitoring and diagnostic procedures; therefore, the refinement of non-invasive methods is of great significance for human application [22, 23]. But, for preclinical testing, the combination of both non-invasive and invasive methods could offer the best outcome providing a safe basis for clinical applications.

Animal models are indispensable tools to develop better devices for medical application [24, 25]. The currently available methods still need to be improved by reducing the number of experimental animals and by increasing reliability [26, 27]. Although various animal models have been developed to study osseointegration, there is still a lack of a well-reproducible, relatively inexpensive and reliable model [6, 28]. Particularly, even the currently available ISO guideline [21] for performing preclinical evaluation of dental implant system suffers from a lack of biomechanical testing. The guideline requires only morphological, radiographical, and histopathological assessments but not any functional investigations of osseointegration [21]. This deficiency is clearly due to the lack of reliable, well-developed biomechanical tests for experimental animals.

Most animal models for the investigation of osseointegration were developed without considering the similarity between the bone microstructure of animals and that of human jaw bones. Consequently, the thereby achieved biomechanical characteristics may be inappropriate since there are remarkable differences between animal and human bones [29]. To approach this problem, we searched for a massive, cortical and spongious bone compartment in rats, suitable for supporting the titanium implants. We found that caudal tail vertebrae were constituted of abundant spongiosa, which presented a similar alveolar structure as the human mandible. Furthermore, the bone marrow parenchyma is also absent in the tail vertebrae, thus having higher similarity to human jaw bone than the hematopoietic femur of the rat, a commonly used experimental model site [12]. Based on these findings, Blazsek et al. [12] developed a novel experimental model for the evaluation of osseointegration and bone remodeling around longitudinally placed titanium implants in tail vertebrae and proposed to name it “OSSI” (OSSeoIntegration) model. We further elaborated the original OSSI model to enable multiple placements of implants in positions perpendicular to the tail [27]. Although these models are both fundamentally new, they both had serious shortages. In the original model [12], the poorly defined surgical procedure and also the lack of complex evaluation procedure led to quite high standard error during experiments making the model suboptimal to quantitatively characterize osseointegration. In our newer model [27], the transversal positioning of the implant into the vertebral body left only a very small amount of bony structure around the bony bed (i.e., 2-mm-thick bony wall), which prevented us to perform biomechanical testings.

Therefore, in the present study, we aimed to refine our original models to develop a quantitative preclinical screening model for osseointegration of implants with special emphasis on biomechanical evaluations. We hypothesized that in the rat tail vertebrae, osseointegration of titanium implants could be quantitatively monitored by a combination of biomechanical resonance frequency analysis and pull-out test, and by structural micro-CT and histomorphometry methods. We found that all of these test systems were applicable for the evaluation of the implant osseointegration process. However, the simultaneous application of these measurement methods and a combined evaluation based on the obtained data were much more advantageous to provide highly reliable and reproducible outcome using a limited number of small experimental animals.

## Materials and methods

### Animals

A total of 63 male Wistar rats (Crl(Wi)Br, Charles River; 450–550 g) from the breeding colony of Semmelweis University were used. They were kept in light-controlled, air-conditioned rooms before and after the surgery in individual stainless steel cages. The surgical procedure was carried out in a specialized operating room.

Since we did not have preceding data with the presently developed methodology, for sample size calculation, we used the pull-out evaluation data at the 4th and 8th week endpoints. At these endpoints, we had 14 animals per group. Then, we used the G*Power free software (University of Dusseldorf, http://www.gpower.hhu.de/en.html). The α-error probe was 0.05, the power was 0.8, the allocation ratio N2/N1 was 1, and the effect size was counted as 2.87. Based on this calculation, we applied sample size *n* = 7 in consecutive experiments.

### Mini-implant design (Fig. 1)

**Fig. 1:**
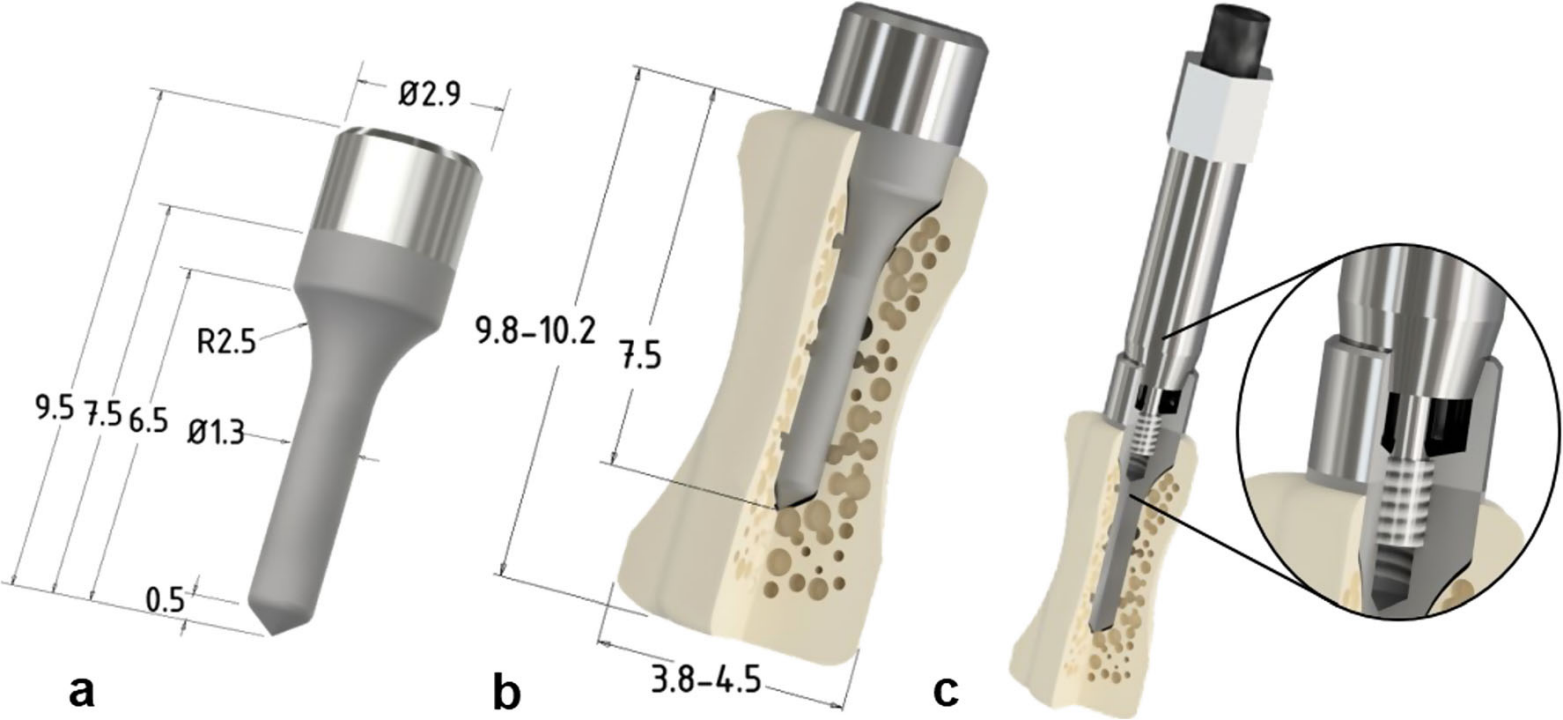
Schematic and real-size illustration of the customized implant and its insertion in the hosting bone. **a** A drawing of the implant. **b** Schematic illustration of the implanted titanium device with the bone. **c** Cross section of connected Smart peg Type 4 with the customized implant

Newly designed and fabricated implants were used during experiments with the consideration of the bony tissue volume of the vertebrae (FullTech Ltd., Hungary). The implants were cylindrical in shape without threads and were made of biocompatible Grade 4 commercially pure titanium (cPTi), fabricated using a CNC lathe machine (EMCO Turn 325, Siemens Ltd., Germany). As we previously reported, the size of the caudal vertebrae of 450–550-g rats was from 9.8 to 10.2 mm in length, and from 3.8 to 4.5 mm in diameter [27]. Accordingly, the implants were set at 2.9 mm in diameter at the level of the neck, and 1.3 mm at the body part. The length of the entire implant was 9.5 mm (Fig. 1a). The entire implant had parallel walls and cylindrical shape without threads. With such a design, we aimed to develop a shape for the pure evaluation of biological integration without any additional influence of the geometrical design (threads, holes, self-tapping). The cylindrical shape allowed us to measure the strength of the anchorage of bone to the titanium and exclude the influence of the implant’s form and standardly monitor osseointegration by biomechanical and structural tests.

All the titanium implants were uniformly modified by sand-blasting (Korox 250, Bego) and subsequently by chemically etching with 43% ortho-phosphoric acid. The geometry of the implant neck was created in such a way that it should to allow a direct connection to SmartPeg® (type 4), the magnetic transducer of the Osstell ISQ device (Osstell AB, Gothenburg, Sweden). Special attention was paid to the proper connection between the implant neck and the SmartPeg® so as to generate reproducible, and quantitative ISQ values. Thus, we followed a similar strategy to that previously reported for testing the stability of mini-implants utilizing the RFA method [30]. The implant neck had an inner thread, which, in the first place, hosted the SmartPeg® during implant stability quotient (ISQ) measurements (Fig. 1c). Then, the SmartPeg® was unscrewed and replaced by a specifically designed hook that served as a stable connection between the pull-out device and the implant during extraction force measurement.

### Surgical procedure

**Fig. 2:**
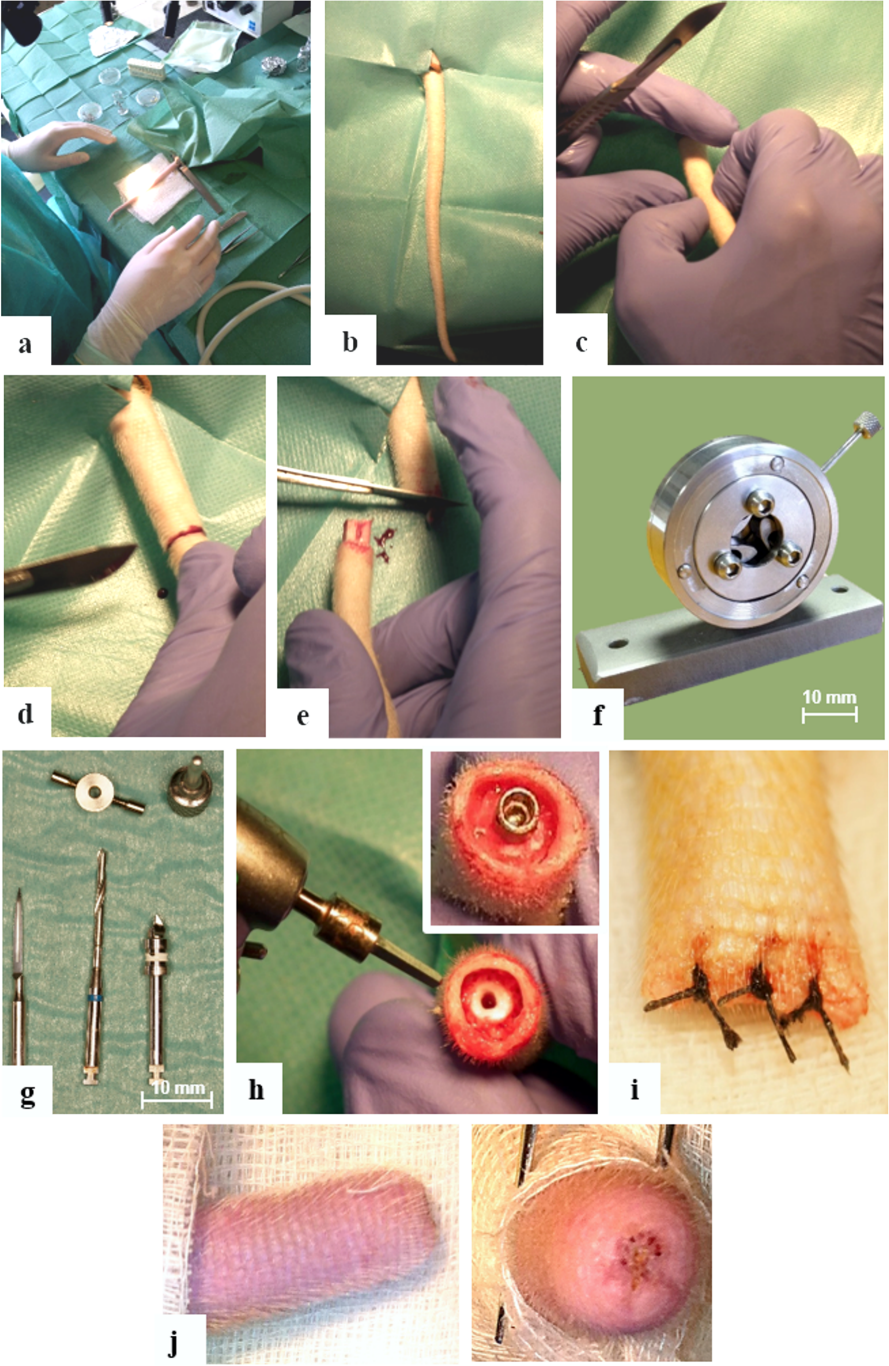
Surgical and postoperative workflow of the preclinical screening model in the rat tail. **a** Surgical setup for the rat tail operation. **b** The cleaned, surface treated and double ligatured rat tail before surgery. **c** The palpation of the intervertebral joints and planning the first incision which is done cutting only the skin. **d** Skin incision 3 mm distally from the C4-C5 vertebrae joint. **e** Dissection of the C4-C5 intervertebral joint in bloodless conditions because of the due to double ligature shown in insert **b**. **f** Surgical guide for standard cavity preparation. **g** Surgical drilling kit for preparation of bony bed. **h** Preparation of the hosting tissue and inserted titanium devices in the C4 rat vertebra. **i** Wound closure of the amputated rat tail after implantation. **j** The amputated tail after 8 weeks of healing, horizontal and vertical views

The surgical procedure is based on our previously published model [12] with a number of important modifications. All the operative procedures were performed in sterile conditions using sterilized equipment, surgical hand pieces, and physiodispenser (Fig. 2), similar to human surgical procedures. The rats were operated under general anesthesia with sodium pentobarbital (Nembutal, CEVA, France, 40 mg/kg body weight, i.p.). The animal was covered by a sterile tissue “barrier” (Mölnlycke®, Sweden), only the surgical field of the tail remained exposed. The weight of animals was registered before and after surgery. First, the tails were mechanically cleaned with warm water and a detergent; second, they were washed in three steps with a disinfectant solution (Softasept, B-Braun) for 3 min each. To control bleeding, double ligatures were positioned at the beginning of the tails. The skin surface of the entire tail was treated with 10% povidone-iodine (Betadine, Egis, Hungary). Three millimeters distally from the C4-C5 vertebrae joint, a circular incision was made and the skin was retracted. With a new blade, the distal part (after C4 vertebra) of the tail was amputated 3 mm proximal to the skin incision.

After the amputation of the distal part of the tail, an axial cavity was made in the opened surface of the C4 vertebra to host the implants using specially designed and fabricated drills (pilot, twist drill, and neck drill) (FullTech Ltd., Hungary) (Fig. 2a) and drilling protocols. We used a surgical guide to facilitate repeatable, reliable, and independent placement of implants (Fig. 2b). The first drilling was performed with the pilot drill to drill through the compact bone with a diameter of 0.5 mm. Then, a twist drill with a diameter of 1.3 mm was used to create a 9-mm deep cavity under water cooling with the support of the surgical guide to make implant placement repeatable in the same position. Finally, the neck drill was used to prepare the space for the implant neck, also with the help of the surgical guide. Implants were placed using an implant driver into the prepared bony cavity. After implantation, the soft tissues were repositioned and the wound was closed using standard non-resorbable 4.0 atraumatic sutures (Dafilon, B Braun). Then, the skin was disinfected with 10% povidone-iodine and the amputated end of the tail was covered with tissue adhesive strips (SteriStrip, 3M). Animals were kept at 32 °C until awakening. No lethal complications happened during the surgery or afterwards.

### Postsurgical treatment

Wound healing was monitored every day during the first week and twice during the second week after surgery. Two types of antiseptic solutions were applied on the surface. Tails were disinfected using 3% hydrogen peroxide solution (Hyperol, Meditop) and then 10% povidone-iodine. Direct palpation of the tail was done for the detection of any inflammation or other changes. A massage of the tail was performed during palpation to stimulate local blood circulation during the first 3 postoperative days.

### Sample harvesting

The animals were sacrificed under general anesthesia with sodium pentobarbital (Nembutal, 40 mg/kg body weight). We sacrificed 21 animals after 4 weeks, 21 animals after 8 weeks, 7 animals after 12 weeks, and 14 animals after 16 weeks. The samples were used either for biomechanical (RFA and pull-out test) or for structural (micro-CT and histomorphometry) analysis. The tail was ligated at the bottom to control bleeding; then, C3-C4 vertebrae were separated from the tails through surgical cutting the joint between C3-C2 vertebrae. The C3 vertebrae were used as healthy controls for C4 in histomorphometrical and micro-CT analyses. For micro-CT, the soft tissues were removed and the vertebrae were kept in 0.9% NaCl solution at 2 °C until evaluation. Samples for histomorphometry were fixed in 10% buffered formaldehyde solution.

**Fig. 3:**
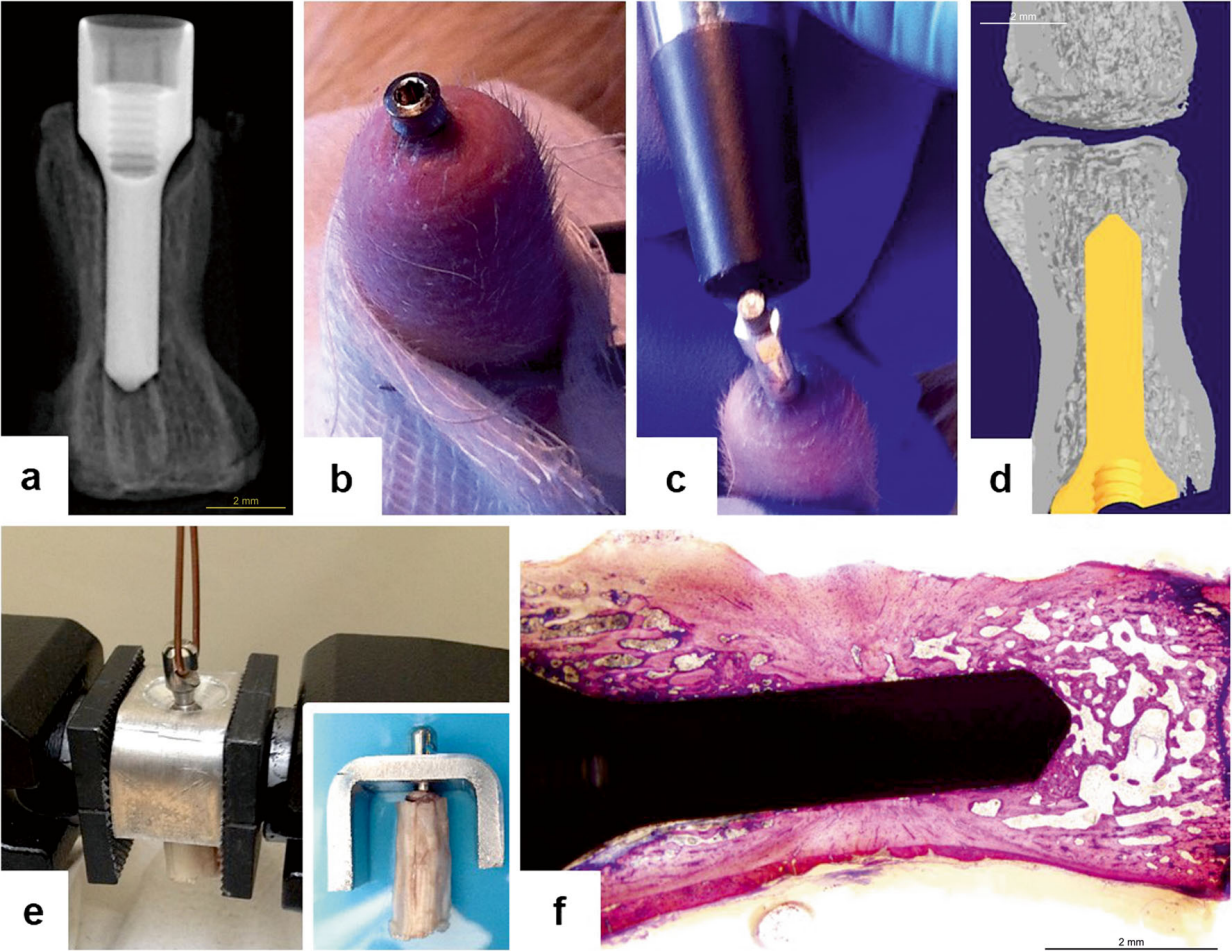
Evaluation of the preclinical screening model in the rat tail. **a** Postoperative control X-ray for midterm evaluation of the healing process. **b** Individually fabricated healing abutment after implant opening. **c** In situ evaluation of implant stability during the healing with smart peg during the healing and the healing cap placed afterwards. **d** Micro-CT capture of the implant within the bone. **e** Axial extraction force measurement device also showing sample positioning. **f** Histomorphometric slide prepared for analysis

We set a complex evaluation protocol to analyze the interosseous implant anchorage in the bony tissue using combined biomechanical and structural methods. The biomechanical evaluation of osseointegration was performed applying RFA and pull-out tests, both on the same samples (Fig. 3). The structural analysis was carried out by micro-CT and histomorphometry using the same samples.

### Biomechanical evaluation

The two biomechanical tests were completed on the day of harvesting. We first performed the resonance frequency analysis, followed by the pull-out test. Fourteen animals were tested at the 4th and 8th weeks, while seven animals were evaluated at the 12th and 16th weeks.

*Resonance frequency analysis* has been recently introduced to dental implantology as a non-invasive method to assess implant stability. It has been claimed to be a simple and reliable evaluation approach for testing implant stability in humans [31, 32]. RFA is based on measurements of the implant stability with a magnetic transducer (SmartPeg®), which is directly connected to the implant head. The transducer is stimulated by electromagnetic waves of the probe (created by the coil in the probe) of the Osstell ISQ device. By sending a magnetic impulse from the probe, the apparatus automatically switches to a mode for detection of resonance frequencies from the SmartPeg®. Based on the level of resonance, the Osstell device produces an ISQ value in the range of 0–100, where larger ISQ values indicate higher stability. To standardize the method in the rat vertebra model, calibration measurements were performed using a calibration block provided by the manufacturing company, Osstell AB. To perform the actual tests, the dissected specimens were fixed by forceps to the surface of the operating table to eliminate micromovements. The SmartPeg® type 4 was screwed gently into the inner thread of the implant neck until reaching resistance. The stability of each implant was recorded in four perpendicular directions, five times per direction. Then, the average of these 20 ISQ values was used to describe the stability of the particular implant. These measurements showed very small standard error suggesting that RFA tests provide reliable values.

#### Pull-out test

The axial extraction force was also used to evaluate the bone anchorage strength to the implant surface. With a specially developed hook screwed into the implant head, we were able to measure the peak force, which was needed to destroy the established biological integration of the titanium implant into bone. The peak force and the instant force as a function of the implant displacement in axial direction were detected using a tensional test machine Instron 5965 (Instron, Norwood, MA, USA). Measurements were done according to the following steps: (a) the hook-head was screwed to the end of the implant. Then, a thin stainless steel cable (Ø1.5 mm) was pulled through the hook-head to provide an appropriate grip for the measuring device; (b) the vertebra was fixed in a metal bracket by self-positioning in the pulling direction and the instrument was balanced, the implant was steadily pulled along the vertical axis until complete extraction; (c) the maximum axial pull-out force of the implant was calculated from the recorded load-displacement curve. The maximal pull-out force (N) represents the strength of osseointegration. The pull-out test was applied in accordance with ASTM F543 (American Society for Testing and Materials, Standard Specification and Test Methods for Metallic Medical Bone-Screws). Its Annex A3 contains directives for the determination of pull-out test measurement parameters.

### Structural analyses

Twenty-one specimens (*n* = 7 animals per group) were used for structural analysis such as micro-CT and histomorphometrical analysis.

#### Micro-CT analysis

Before histological testing, we performed a 3D radiographic data acquisition to detect the structural basis of implant stability in the reconstructed 3D images (SkyScan 1172, Kontich, Belgium). The device has an X-ray source from a sealed micro focus X-ray tube with a spot size of 8 μm. In the present work an Al+Cu filter (Al 1 mm and Cu 0.05 mm) was used. Recovered implant samples with bone were scanned at 360° rotation at 0.3° rotation step at 80 kV, 124 mA, 4598 ms exposure time with an isometric voxel size of 12 μm. For the reconstruction of raw images, a cone-beam volumetric algorithm was used with the NRecon V1.6.10.1 version software (SkyScan, Kontich, Belgium). Measurements were performed within a certain region of interest (ROI) in the reconstructed images using the software CTAn, version 1.14.4.1+ (SkyScan, Kontich, Belgium) [33]. The described protocol for scanning and reconstruction was designed and optimized to our experimental conditions in order to overcome the x-ray scattering on the metal surface.

The scanned samples were evaluated in 2D and 3D perspectives with task lists developed for this purpose in the CTAn software. The calculated intersection surface/tissue surface ratio (i.S/TS) was used in 2D analysis for characterizing the bone to implant contact. Based on the manufacturer’s instruction (SkyScan 1172, Kontich, Belgium) and our calibration process, we chose the 12-pixel wide dilation length around the implant for determining the intersection surface value expressed in percentage. For the bone volume assessment, a 38-voxel (0.461 μm) thick cylindrical volume of interest was selected around the titanium implant [33–35]. The manual global threshold method was used for the segmentation of new bone visualization. For determining the percentage of bone volume value, bone volume/tissue volume ratio was calculated (BV/TS).

#### Histology and histomorphometry

**Fig. 4:**
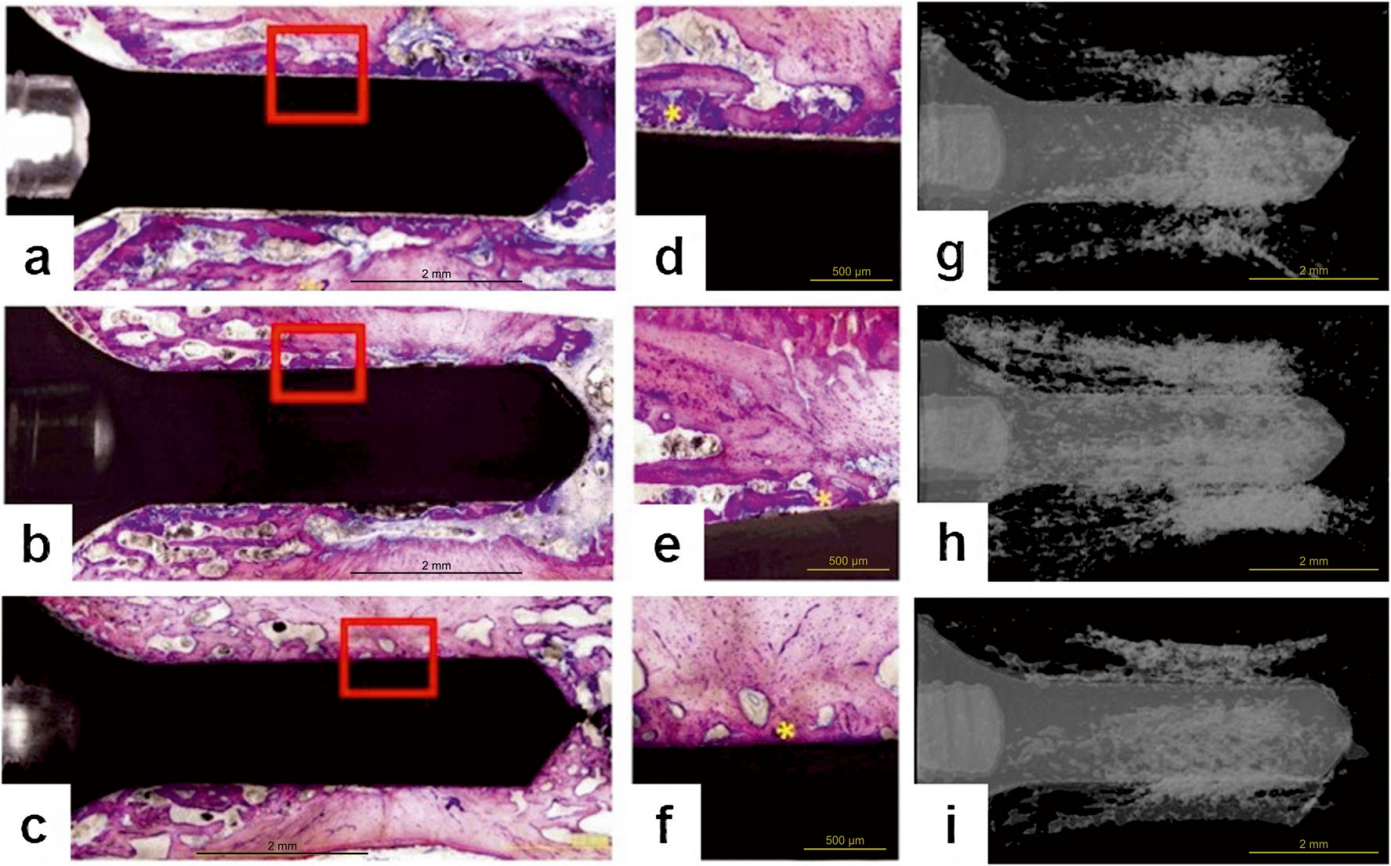
Histological slices and 3D rendered images from different healing periods. **a** Histomorphometric slide at the 4th week of healing: active bone remodeling is taking place (× 1.25). **b** Histomorphometric slide after 8 weeks of healing: newly formed bone has a higher intensity of staining due to the lower mineralization rate of the bone compared to the matured one. Bone density is lower compared to the 4th week and 16th week of healing (× 1.25). **c** Histomorphometrical slide at the 16th week of healing: bone regeneration and bone to titanium surface have reached biological equilibrium. We did not detect any higher intensity of staining due to the stabilized remodeling process (× 1.25). **d** High percentage of smear layer is presented 200 μm around the implant at week 4 (× 10). **e** A lower rate of debris is found at week 8 suggesting the progress of new bone formation (× 10). **f** Well-formed direct bone to implant contacts is present. The biological equilibrium is reached at the 16th week (× 10). **g** At the micro-CT image from 4 weeks of healing the implant is surrounded by smear layer in approximately 200-μm thickness. **h** Micro-CT image after 8 weeks of healing shows newly formed bone at the same location as observed on the histological slide. Bone density is lower than corresponding values at week 4 and week 16. **i** Micro-CT image at the 16th week of healing shows bone regeneration: bone to titanium surface have reached biological equilibrium

After micro-CT measurements, the samples were chemically fixed and embedded as previously reported [36]. The implants were then cut with a diamond saw along the longitudinal central axis of the implants. The slices were then mounted on Plexiglas boards and surface-stained with McNeal’s Tetrachrome, basic Fuchsine and Toluidine Blue O [37] for histomorphometric analysis. The bone-implant contact (BIC %) was then analyzed under light microscope with × 10 magnification (Fig. 4).

### Statistical analyses

One-way ANOVA test and Tukey-Kramer Multiple Comparisons post hoc test were used to evaluate the statistical significance between the different endpoints of healing using RFA, pull-out test, micro-CT, and histomorphometry analysis. Each data from each healing period were compared to each other. For the evaluation of correlations, Spearman’s test was applied to seek interrelationships between pull-out and RFA biomechanical tests and also between micro-CT and histomorphometric structural analyses, respectively. Each statistical test was performed in Statistics 12 software (StaSoft, Inc. USA).

## Results

### Biomechanical evaluation of osseointegration of implants into tail vertebra

**Fig. 5:**
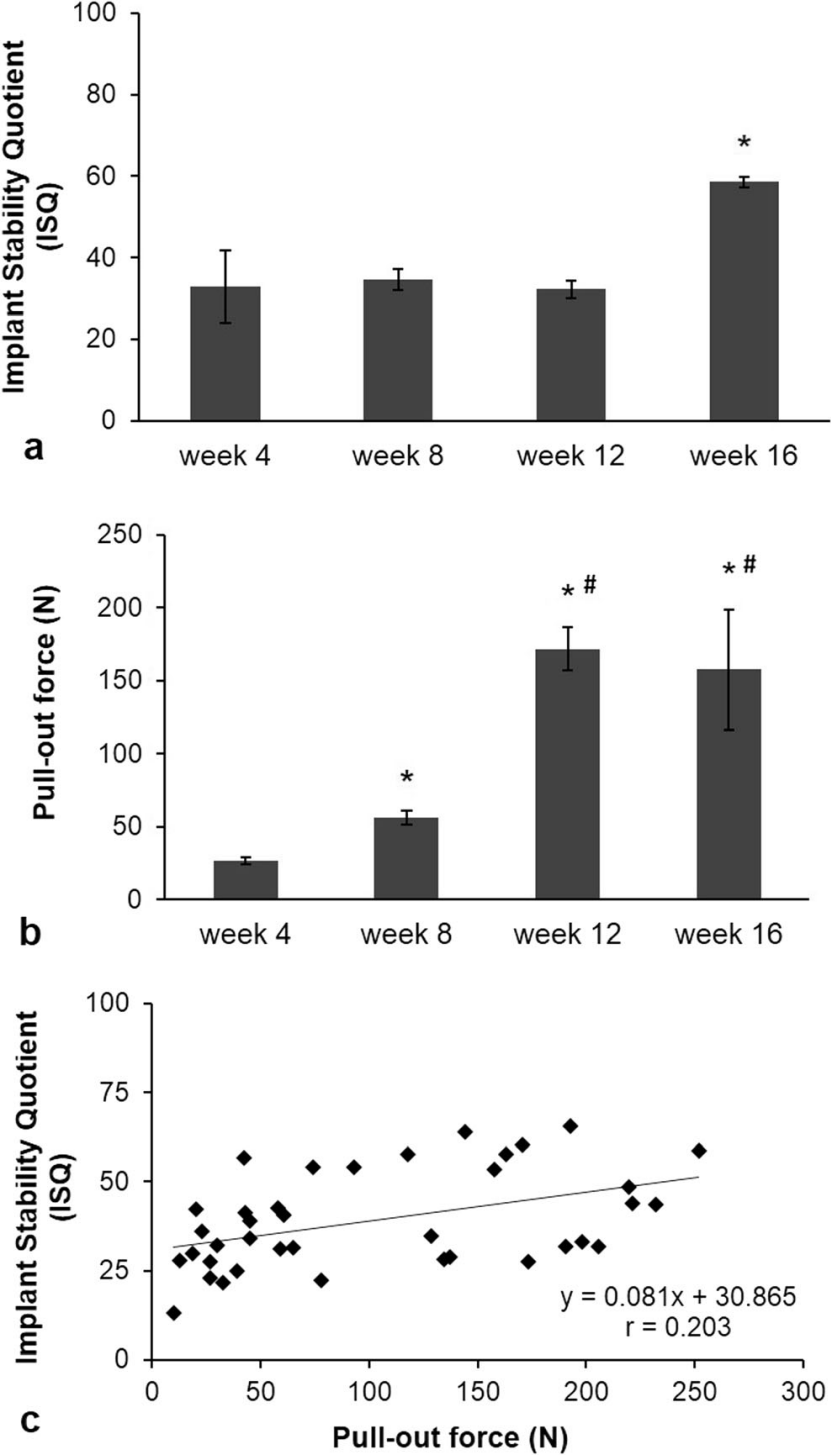
Comparison of pull-out test and resonance frequency analysis as measures of osseointegration at different time points during healing. **a** Evaluation of titanium devices stability using RFA at week 4, week 8, and week 16 after implantation in rat tail model. Mean ± SEM. *p < 0.05 vs. week 4 and vs. week 8 and vs. week 12 results. **b** Evaluation of titanium devices stability using pull-out test at week 4, week 8, and week 16 after implantation in rat tail model. Mean ± SEM. *p < 0.05 vs. week 4; ^#^*p* < 0.05 vs. week 8 results. **c** Correlation analysis between non-invasive (RFA) and invasive evaluation (pull-out test) methods of implant stability (*r* = 0.202)

ISQ values moderately changed in the initial healing time. A significant, 1.6-fold increase of ISQ values occurred from week 4 (37 ± 4) to week 16 (60 ± 3) (Fig. 5a). However, no significant difference was observed between values corresponding to healing periods week 4, week 8, and week 12. The pull-out force significantly increased with time and reached a plateau at the 12th week postoperatively (Fig. 5b). The high sensitivity of this test was demonstrated by the fact that the pull-out force increased by approximately 500% between week 4 and week 12. There was no further significant change in this parameter between the 12th and 16th weeks.

**Fig. 6:**
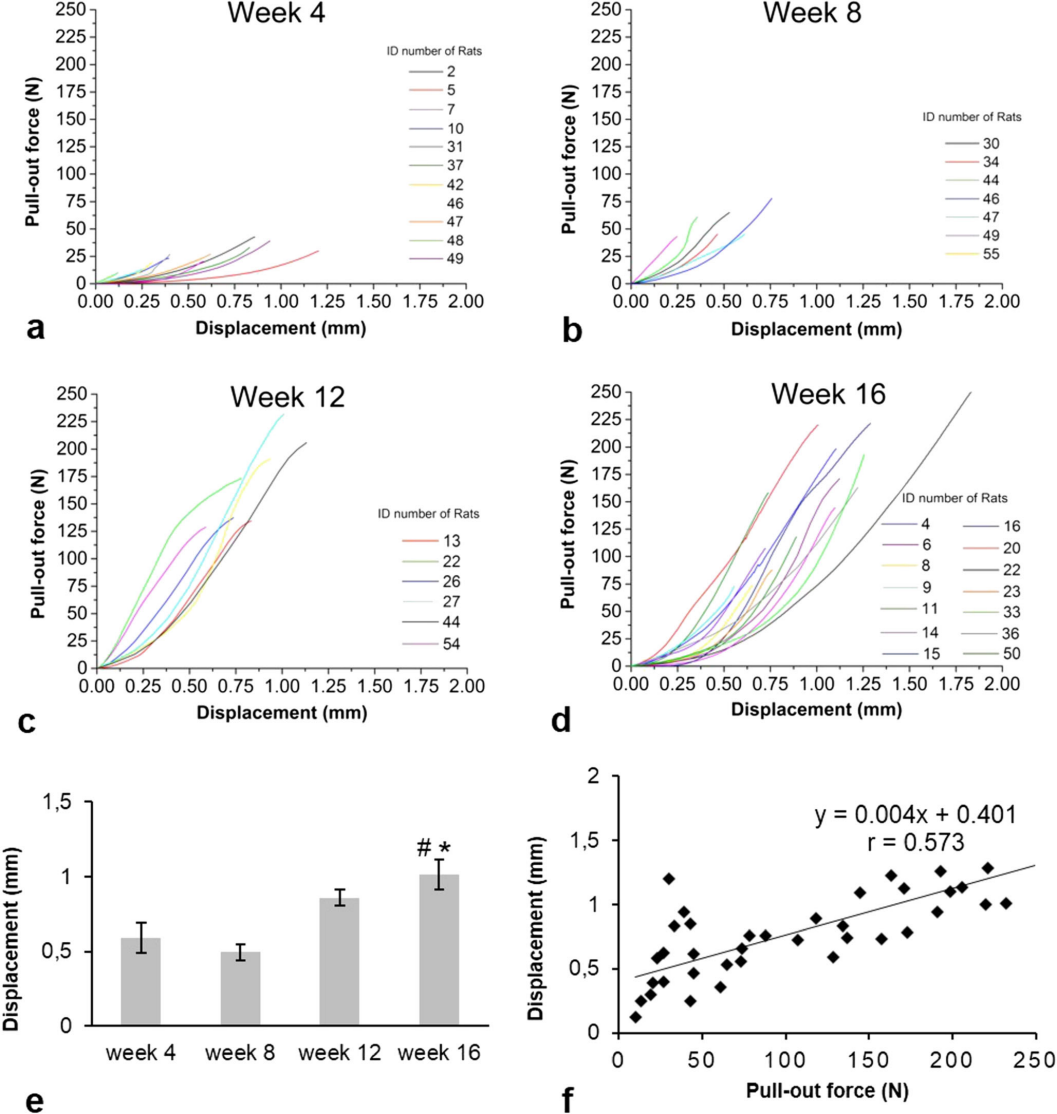
Pull-out test curves (force as a function of displacement) at different time points during healing. Week 4 (**a**), 8 (**b**), 12 (**c**), and 16 (**d**) curves following implant placement show how osseointegration influence peak force value and the slope of the curve. The higher the connection stiffness (according to healing time) among the implant and bone, the higher the force needed to destroy it. The displacement of the implant during the test gradually increased. **e** Evaluation of titanium implant displacement during pull-out force measurements on week 4; week 8, week 12, week 16 after implantation in rat tail model. Mean ± SEM. *p < 0.05 vs. week 4 results; ^#^*p < 0.05 vs. week 8 results. **f** A strong positive correlation is found between pull-out force and displacement peak values

In Fig. 6a–d, the curves demonstrated how healing time influence osseointegration factors such as peak force, displacement value, and curve slope (Fig. 6a–d). The displacement values started to increase at week 12, reaching significantly elevated level only at week 16 compared to week 4 (Fig. 6e). When all relevant data were depicted in a single graph, a positive correlation (*r* = 0.573) was found between the pull-out force and the displacement values (Fig. 6f).

Since no clear data were available about the meaning of ISQ unit of the Osstell ISQ device in the literature, we attempted to translate it to real physical force by correlating the ISQ values to the corresponding pull-out forces. The correlation analysis showed only a moderate relationship (*r* = 0.203) (Fig. 5c). This was primarily due to the relatively low sensitivity resonance frequency test over the pull-out test as described above.

### Structural analysis of osseointegration of implants into tail vertebra

**Fig. 7:**
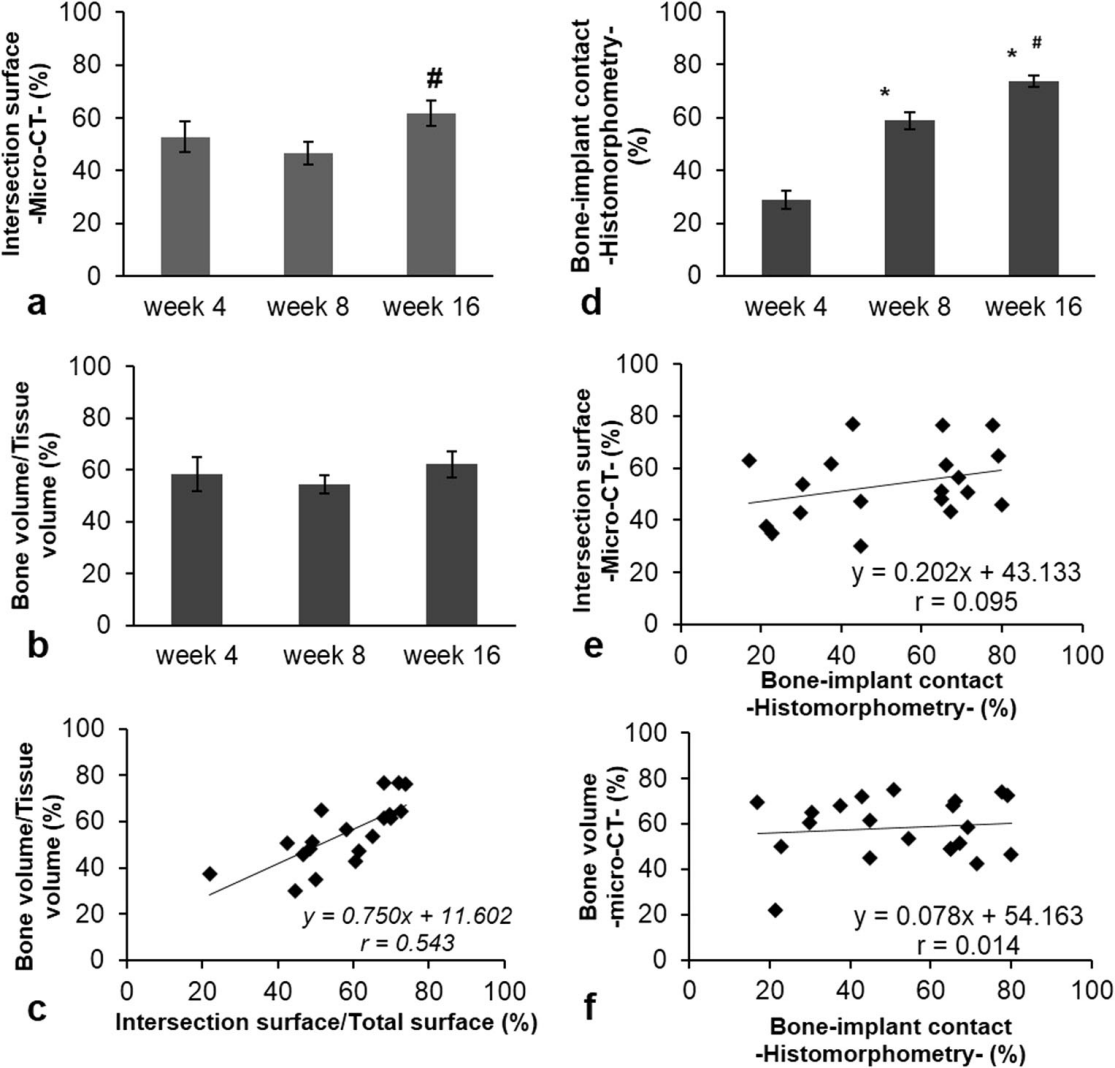
Comparison of histomorphometry and micro-CT analysis as measures of osseointegration at different time points during healing. **a** Evaluation of implants stabilities using 2D analysis of the micro-CT data presented in i.S/TS (intersection surface) at week 4, week 8, and week 16 after implantation in rat tail model. Mean ± SEM. *p < 0.05 vs. week 4 results. **b** Evaluation of bone volume around titanium implants using 3D analysis of the micro-CT data presented in BV/TV at week 4, week 8, and week 16 after implantation in rat tail model. Mean ± SEM. *p < 0.05 vs. week 4 results. **c** A strong positive correlation was observed between BIC evaluated by micro-CT and BV/TV. **d** Evaluation of titanium devices stability using histomorphometric analysis measuring the BIC ratio at week 4, week 8, and week 16 after implantation in rat tail model. Mean ± SEM. *p < 0.05 vs. week 4 results; ^#^*p* < 0.05 vs. week 8 results. **e** Correlation of BIC ratio values measured by micro-CT and histomorphometry. **f** No correlation was found between of BV/TV and histologically evaluated BICs

The 2D analysis results of micro-CT scans showed that the i.S/TS values were 58%, 48%, and 61% at weeks 4, 8, and 16, respectively. Statistically significant difference (*p* < 0.05) was observed between the 8th and 16th weeks (Fig. 7a). The unexpectedly high i.S/TS values obtained at the 4th week after surgery were due to the high level of remaining debris between the implant body. As it turned out, the x-ray absorption of the debris was nearly the same as that of the vertebral bony tissue Indeed, individual images showed that at week 4, an approximately 200-μm-thick homogenous debris layer covered almost the entire surface of the implant (Fig. 4g). At the 8th week, this coverage around implants was interrupted as the smear layer gradually disappeared (Fig. 4h). Finally, at week 16, no debris was seen in the images (Fig. 4i).

In the 3D evaluation, BV/TV values were 58%, 56%, and 61% at 4, 8, and 16 weeks respectively (Fig. 7d). No significant differences were found between the groups in BV/TV results. A positive correlation was found between BV/TV and i.S/TS data (*r* = 0.544) in bone micromorphometric results (Fig. 7c). This correlation indicated a relationship between intersection surface coverage of the bone and bone volume/tissue volume values in individual specimens.

At the 4th week, a low level of real BIC was detected corresponding to 28.55 ± 3.54% coverage of the interface by histomorphometry (Fig. 7d). The interspace between bone tissue and implants was largely filled with bone debris (Fig. 4a). In comparison with week 4, BIC values (61.66 ± 3.31%) increased significantly at week 8 (*p* < 0.05) (Fig. 7d), with only sporadically visible debris around the implants (Fig. 4b). At the 16th week, BIC values further increased to 73.85 ± 2.12% (Fig. 7d) (*p* < 0.05 vs week 8) with no visible debris (Fig. 4c). Regular trabecular bone was seen surrounding the implants (Fig. 4d–f). These data indicated that BIC sensitively reflected the progress of osseointegration with time during a 16-week-long experimental period.

There was no correlation between BV/TV and the histomorphometrical BIC results (*r* = 0.014) (Fig. 7f). However, a weak positive correlation was detected between i.S/TS and BIC (*r* = 0.096) (Fig. 7e).

## Discussion

Functional tooth replacement and bone regeneration are parts of the daily practice of modern dentistry, but a well-reproducible and relatively inexpensive preclinical functional test system is still missing. We aimed to develop a new quantitative animal screening model for the osseointegration of implants with special emphasis on biomechanical evaluations. In the rat tail vertebrae, we monitored the osseointegration of titanium implants quantitatively by a combination of RFA, biomechanical pull-out test, micro-CT, and histomorphometric methods. We observed that these test systems are individually applicable to the evaluation of the implant osseointegration process. But the simultaneous application of these methods and a combined evaluation are much more advantageous for the screening process to provide a highly reliable and reproducible outcome using a limited number of experimental animals. Our approach is particularly important as a biomechanical investigation; since the present ISO guideline for preclinical evaluation of dental implants suffers from complete lack of biomechanical testings [21], it is particularly important to set up and standardize such methods.

Our present work offers considerable upgrade over our previously published data [12] which introduced longitudinal implant placement into the vertebral axis. The high variability of the previous results was primarily due to fact that cylindrical cavity for implantation was 1 mm wider in diameter than the size of the implant thereby creating an empty space around the implant. Only the very tip of the implant was connected directly to the bone. Additionally, the surgical procedure was poorly defined [12]. In the present work, the prepared implant bed had exactly the same size as the implants. Furthermore, implants were prepared with parallel wall with no threads to monitor biological bone bonding without the modifying effects of threads and various strengths of thread fixation. During implant placement, hand-free drilling always decreases the accuracy of the process even for experienced surgeons [38]. Consequently, the application of the surgical guide which we developed and described above greatly increased the accuracy and reproducibility of drilling position in the very center of the vertebra, perpendicular to the vertebral end-surface. Finally, we developed a postsurgical infection-preventing protocol. All these modifications together yielded a well-defined preclinical model having minimal complications in experimentation and very low variability in the data obtained [12].

Our results showed that the most sensitive and reliable preclinical osseointegration test was the pull-out test. This method has high sensitivity to small and dynamic changes in the implant-bone interface. The data received by the pull-out measurement had small standard error, which suggested that the biological processes were quite uniform in various animal species [39–41]. The disadvantage of the pull-out test is that it is an invasive method [42]. Therefore, it is suitable only in preclinical studies. Previous studies showed very divergent outcome. As it appears in studies using non-threaded implants [43–45], experiments showed that the pull-out test is a very reliable method, yielding a steep increase of extraction force with time. However, the pull-out technique is not suitable to the direct determination of osseointegration using commercially available dental implants since those are always threaded, as secondary stability is highly masked [13, 42, 46]. To avoid this problem, simple test bodies (e.g., discs) have been developed [47–49]. But the validity of these results was limited, since test bodies were inserted into the cortical bone and fixed with a pre-shaped titanium band that bore pressure on the samples and affected healing [47–49].

In our experiments, displacement values started to increase at week 12, reaching a significantly elevated level by doubling at week 16 compared to week 4. These results are in line with the few previous observations, showing that this can be used as osseointegration detection when considerable changes happen [43, 44]. We also analyzed the force as a function of displacement showing the mechanical energy needed to destroy the new bone around implants. Obviously, shortly after implantation, osseointegration is weak, and force-displacement curve shows that small displacement, at an early stage, can destroy developing bone connection. Osseointegration improves with the healing time, due to the increased BIC ratio. Our results are in line with previous studies [43, 50] showing increasing elasticity of bone-implant connection as well. With time, the implants have higher resistance to displacement before the destruction of bone-implant connection. Our data show that implant displacement is in the range of 0.5–2 mm.

The RFA has been successfully used in clinical studies as the only non-invasive, functional measurement method. It is regarded as a sufficient tool for evaluating the course of intraosseous implant stability in clinical practice [9, 51–53] and in preclinical settings [54–58]. We found that ISQ values moderately changed with healing time. Increases in ISQ values showed a significant level at week 16. However, differences fell short of significance at weeks 8 and 12. Other in vivo studies involving RFA evaluation are in accordance with our findings. We observed that ISQ values doubled between weeks 4 and 16. A similar magnitude of increase in ISQ values were also previously observed in experiments applying similar timeframe in various species including humans [9, 55, 57, 58]. These results of preclinical studies are contradictory. Some of the studies did not detect any change during healing between primary and secondary stabilities with RFA [59, 60]. In contrast, other results showed a dynamic increase in ISQ values from primary to secondary stability [9, 54–58]. Taken together, RFA is an appropriate method for determining differences between very early and late stages of osseointegration. But it is not sensitive enough to detect minor changes between relatively close time points during the course of the osseointegration process. Therefore, it is a useful technology but only for well reproducible preclinical screening. Other methods, such as the pull-out tests, should also be used in parallel.

The correlation analysis showed that there was a correlation between ISQ values and pull-out results, both increasing with time, but the fitted line is very low. The pull-out test gives real physical values in Newton while the RFA test provide only unidimensional relative values. However, more importantly, we observed a fivefold increase in pull-out values over time with minimal standard error versus moderate, i.e., only 50% increase in ISQ mean accompanied with high standard error. The simultaneous application of both methods is important, because they together provide a good estimate of osseointegration in preclinical research. Additionally, the more sensitive pull-out test cannot be used in clinical situations since it is invasive. Nevertheless, our present results show that the ISQ values provide reasonable functional estimation, although to a lower extent than pull-out. Therefore, they can be used as a functional osseointegration test when combined with other, more sensitive methods.

Histomorphometric images showed that the interspace between bone tissue and implants was largely filled with bone debris at week 4, with reduced debris at week 8, and no debris at week 16. As debris can be well differentiated from real bone implant contact by histomorphometric analysis [19], it revealed a more than 140% increase in BIC values. This is in line with multiple preceding studies, and also the related ISO guidelines, suggesting that BIC analysis is the best available non-functional method to evaluate osseointegration [20, 21] .

On the contrary, the 2D analysis of micro-CT scans yielded less convincing results. Statistically significant difference in i.S/TS values was observed only between the 8th and 16th weeks. At the 4th week, the high level of remaining debris between the implant body and the bony bed masked the relative low contact between bone and implant. At later time points, debris-caused background decreased, while real bone-implant intersection areas increased, finally resulting in a far more moderate elevation in i.S/TS values than that in BIC values. This is in line with previous observations that bone debris can overshadow real BIC analysis [18, 19, 61, 62].

As we expected, in the 3D evaluation of our work, BV/TV values between the 4th and 16th weeks showed no significant differences between the groups in BV/TV results. The ROI for BV/TV detection was done in a 0.46-μm-wide cylindrical volume around the titanium implant excluding the 12-pixel dilation range around the implant, i.e., in its immediate vicinity. The macro design of the implants affect the architecture of the bone which leads to the active bone remodeling process [63]. When threads applied, primary stability is high, but they create high stress in the surrounding bone area leading to a highly active resorption and considerable remodeling process especially in that with high level of primary stability [63–65]. But we used implants without thread and special care postoperative care prevented local infections [27] also diminishing the necessary remodeling process. Taken together histomorphometry seems to be superior vs both 2D and 3D micro-CT analyses for monitoring osseointegration in our rat tail model.

The clinical relevance of the present work is that it offers a small animal system that is suitable for modeling the osseointegration of various implant materials and surface treatments in an inexpensive, reproducible manner. The rat tail vertebrae have high similarities to human jaw bone. They consist of massive, cortical, and spongious bone compartments, suitable for supporting titanium implants and absent of bone marrow parenchyma [12]. Therefore, misbalances in implant integration leading to peri-implantitis and their possible treatments can also be monitored using this novel osseointegration system. In this model, implant osseointegration may also be studied under various adverse conditions such as diabetes [66, 67], parathyroid dysfunctions [68], and osteoporotic conditions [69]. Collectively, these possibilities can be applied to develop novel preventive and therapeutic strategies that can be then transferred into clinical practice.

Clearly, the present study has limitations. First of all, the presented animal model could be extrapolated to human clinical situations only with great cautions, because of the significant species differences. Second, in the rat tail model, one of the most important components of the oral osseointegration process is missing, and this is the oral microflora. Nevertheless, the data provided by our novel model system may yield valuable preclinical information for the implant osseointegration process, in an inexpensive and reliable manner. These results can be applied then to large animal models and also in clinical trials.

## Conclusions

In conclusion, the present data suggest that the caudal vertebrae osseointegration model is useful for the preclinical evaluation of implant integration to bone. The combination of the biomechanical resonance frequency analysis and pull-out test and the structural histomorphometry and micro-CT methods offers a well-reproducible small animal system which is suitable to study the integration of various implant materials and surface treatments. The described approach also allows to test implant osseointegration success in various health conditions such as age variations, and in various disorders such as diabetes and parathyroid dysfunction.
